# Effects of 5 wt.% Polycaprolactone, Polyhydroxybutyrate and Polyvinyltrimethoxysilane on the Properties of Ag/Zn/Mg Alloy

**DOI:** 10.3390/ma15155421

**Published:** 2022-08-05

**Authors:** Marzieh Rabiei, Motahareh Sadat Raziyan, Reza Ebrahimi-Kahrizsangi, Sohrab Nasiri, Arvydas Palevicius, Giedrius Janusas, Andrius Vilkauskas

**Affiliations:** 1 Faculty of Mechanical Engineering and Design, Kaunas University of Technology, LT-51424 Kaunas, Lithuania; 2 Advanced Materials Research Center, Department of Materials Engineering, Najafabad Branch, Islamic Azad University of Najafabad, Najafabad P.O. Box 85141-43131, Iran

**Keywords:** Ag/Zn/Mg alloy, PolyCaproLactone, PolyHydroxyButyrate, PolyVinylTriMethoxy-Silane, mechanical properties, bioactivity

## Abstract

Mg-based alloys have several suitable properties for biomaterials, but they have major problems of being less antibacterial and have a low mechanical strength. To solve these problems, a new combination of Ag/Zn/Mg was prepared in this study, where the presence of Zn and Ag can help to increase the bioactivity. The use of 5 wt.% polymers consisting of PolyCaproLactone (PCL), PolyHydroxyButyrate (PHB) and PolyVinylTriMethoxySilane (PVTMS) is also investigated. DSC, XRD, TEM, FTIR, SEM, and EDAX analysis, as well as mechanical and bioactive behavior, were investigated to characterize the prepared composites. In the comparison, the best behavior was found when PHB was used. The results show that the strength values ranged from ~201 to 261 MPa.

## 1. Introduction

Composite materials are composed of two or more components, and each component may have certain properties [1]. Due to their good biocompatibility, Mg alloys are potential candidates for use in biotechnology [2]. Taking into account the low mechanical properties of Mg alloys, it is important to reduce the rate of degradation of Mg alloys by various techniques, such as alloying and using appropriate polymers [3]. Generally, the additives are polymers and, usually, polymers that are used to improve mechanical and thermal properties [4]. There are several polymers within whose structures the energetic groups have not been observed. These polymers are introduced as neutral polymers, such as PolyCaproLactone (PCL), PolyHydroxyButyrate (PHB) and PolyVinylTriMethoxySilane (PVTMS). The technology for obtaining bio-metallic materials and biopolymers has been extensively studied in the last decade. The extracted results from composites consisting of bio-metals and biopolymers have attracted attention [5,6]. These bio-composites are designed to provide an optimal combination of mechanical, physical, chemical, and bioactive properties that cannot be achieved with any of their individual components. Recently, metallic biomaterials have been considered, especially silver alloys due to their antibacterial features and biocompatibility [7]. Most metallic alloys, such as cobalt–chrome alloys, titanium alloys and magnesium alloys, each have a low bioactivity ratio, high density and elastic coefficient via free toxic ions. Therefore, using these alloys led to major problems, such as the repulsion of scaffold and tissue. In addition, according to these toxic ions after the first surgery for planting, a second surgery to remove the scaffold is necessary. This matter is not economical, and the possibility of injury to the body is increased [8,9]. Silver is a soft and shiny transition metal that is known to have the highest reflectivity of all metals. It is known that silver is biologically active when dispersed [10]. It has been proven that Zn can improve the mechanical properties of silver, and this element, Zn, has several properties in the application of biomaterials [11]. It has been shown that the use of Zn can lead to a reduction in the crystal size of Mg in alloys, thereby increasing its strength [12]. There are several findings shown to improve Ag/Zn/Mg alloys. For example, Peng et al. prepared Ag/Zn/Mg alloys by selective laser melting to improve their mechanical properties [13]. Zhang et al. investigated the influence of Zn and/or Ag additives on the microstructure and properties of Mg alloys [14]. Zhang et al. studied the effects of Ag on the tensile strength and fracture toughness of novel Mg alloys [15]. Generally, the major problem of using metals as biomaterials is related to the repulsion of tissue. This repulsion causes osteoporosis through the initial piezoelectric feature of bone that, with applying force on the bone, increases the electric field. This electric field causes calcium absorption, but with applying force on the scaffold, the ratio of absorption will be decreased, and the bone will become empty [16,17]. In this study, to solve the problem of low biocompatibility and poor mechanical properties of Ag/Zn/Mg alloy, three bioactive polymers—PCL, PHB and PVTMS—were selected and comprehensively investigated.

## 2. Experimental Methods

### Preparation of Composite Ag/Zn/Mg + PCL:PHB:PVTMS

**Figure 1:**
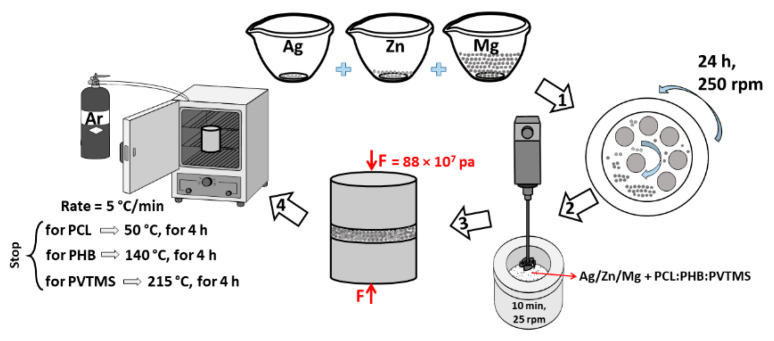
The schematic route for fabricating composites.

The chemical structures of PCL, PHB, and PVTMS are shown in Figures S1, S2 and S3, respectively. In addition, Figure S4 shows the synthesis route of Ag/Zn/Mg alloy. According to Figure 1, for preparing composites: (1) Powder consisting of 3 wt.% Ag/7 wt.% Zn/85 wt.% Mg was considered as matrix, and 5 wt.% PCL:PHB:PVTMS was added as reinforcement before pressing. (2) The alloy and 5 wt.% PCL or PHB or PVTMS were mixed with a mechanical stirrer for 10 min. (3) The powders were placed in the mold, and a few drops of water were added for better uniformity to the pressing process, and cylindrical specimens (with a diameter of 5 mm and a height of 10 mm) were prepared. (4) Based on differential scanning calorimetry (DSC), the temperatures for sintering the samples were set to 50 °C, 140 °C, and 215 °C (4 h) for PCL, PHB, and PVTMS, respectively. Furthermore, the theoretical and experimental values for density and porosity of the specimens are listed in Table 1.

## 3. Results and Discussion

### 3.1. Thermal Analysis of Alloy and Composites

**Figure 2:**
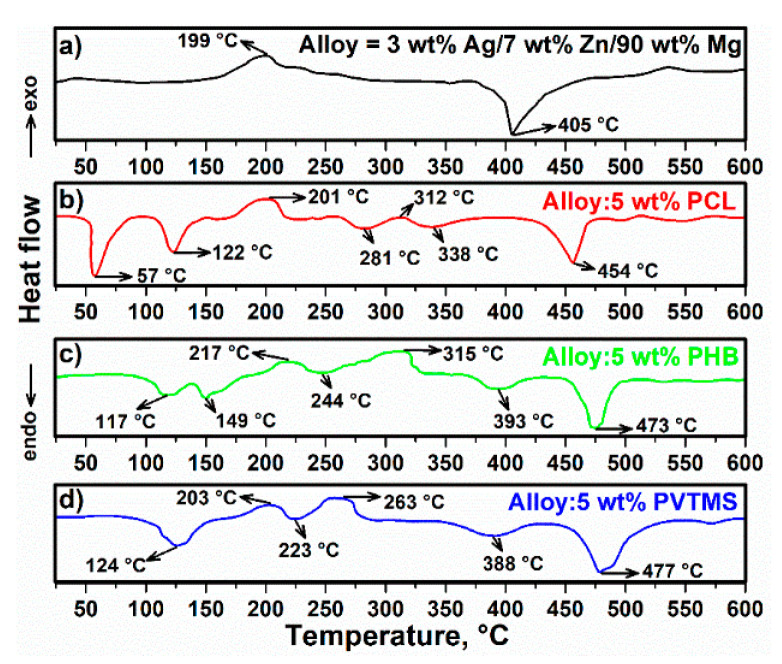
DSC curve of (**a**) alloy, (**b**) C1, (**c**) C2 and (**d**) C3.

The DSC curves of the alloy and composites are shown in Figure 2. In the DSC curve of alloy, the exothermic and endothermic spectra at 199 °C and 405 °C were clearly observed and related to intermetallic phase transformations [15]. Therefore, these temperatures were chosen for heat treatments to produce an Ag/Zn/Mg alloy. The DSC curve of composite C1 is shown in Figure 2b. Moreover, the endothermic peaks at 57 °C and 122 °C are associated with the melting point of PCL and water evaporation inside the structure, respectively [18,19,20]. In addition, the exothermic peaks at 201 °C and 312 °C correspond to alloying and there can be a binary intermetallic transformation between Mg and Ag [21]. An additional endothermic peak at 281 °C is associated with the maximum formation of the metastable MgZn_2_ phase and can also be attributed to the removal of carbamide (C-H-N-O) [22]. In addition, the endothermic peak at ~338 °C indicates the melting of Mg_2_Zn_3_ [23,24]. Moreover, the endothermic spectrum in the range from 425 °C to 500 °C is related to MgZn_2_, and it is possible that one or more reactions occurred within these ranges of temperatures [25,26]. From the DSC curve of C2 (Figure 2c), it can be seen that the water and volatiles evaporated at 117 °C [16]. In addition, the endothermic peak at 149 °C is almost certainly due to the melting point of PHB [27]. Moreover, an exothermic reaction occurred at 217 °C, which can be attributed to intermetallic phase transformation and degradation of PHB [28,29]. Two endothermic spectra were recorded at 244 °C and 393 °C, which are related to the metastable MgZn_2_ and Mg_2_Zn_3_ in tandem [22,23]. From Figure 2d, it is clear that the reaction at 124 °C is related to the evaporated and loosed water [30]. Additionally, the exothermic and endothermic spectra of C3 at 203 °C and 388 °C correspond to the intermetallic transformation between Mg and Zn. It appears that the melting point and decomposition point of PVTMS are 223 °C and 263 °C when the endothermic and exothermic spectra occur at these temperatures. After studying the thermal analysis and melting point of PCL, PHB, and PVTMS, the sintering temperatures of C1, C2, and C3 were set at 50 °C, 140 °C, and 215 °C (below the melting point of the polymers) for 4 h, respectively.

### 3.2. X-ray Diffraction of Alloy and Composites

**Figure 3:**
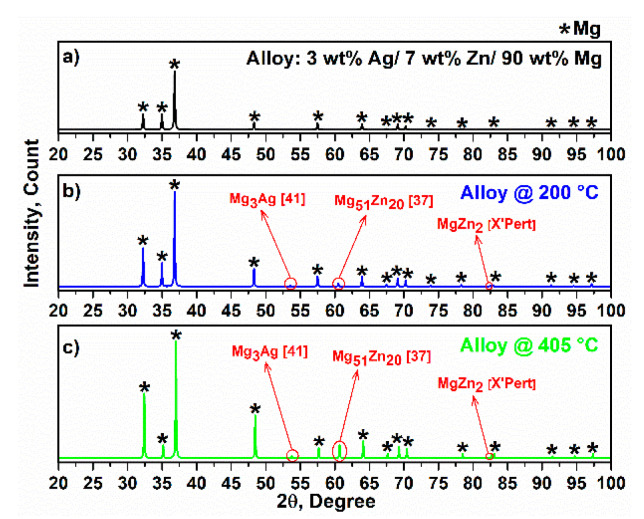
X-ray diffraction of (**a**) alloy and sintered alloy at (**b**) 200 °C and (**c**) 405 °C.

**Figure 4:**
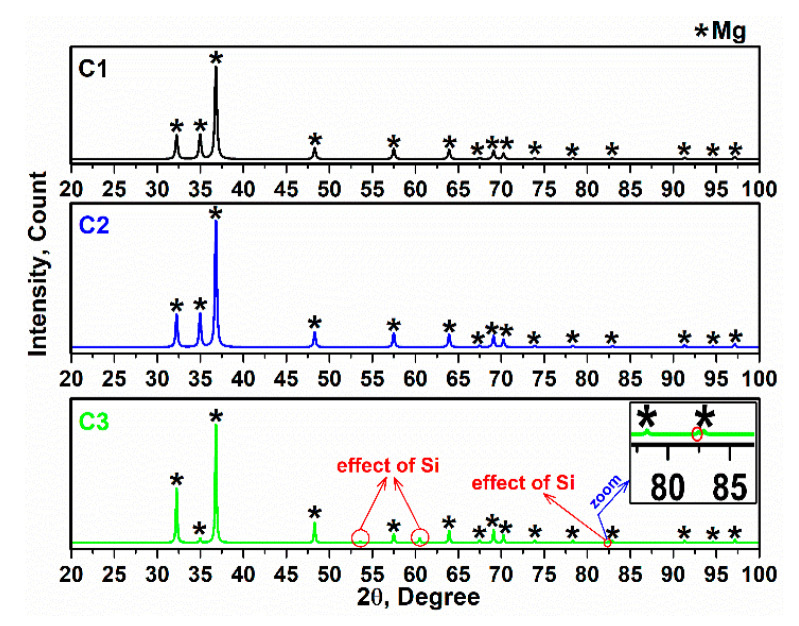
X-ray diffraction of composites.

The X-ray diffraction of alloy and composites are depicted in Figure 3 and Figure 4 in tandem. The XRD pattern corresponds to reference code 00-035-0821 (X’pert) and Ref [31]. According to Figure 3a, the sharp peak appeared at 2ϴ = 36.79° (10$\bar{1}1)$ and is directly attributable to Mg [32]. In addition, the hexagonal lattice parameter values were calculated: a = 0.32094 nm and c = 0.52112 nm, respectively. According to the solubility of Zn in Mg, a ratio of 1.6 wt.% was obtained, and the remaining Zn content of up to 3 wt.% led to the formation of intermetallic phases. Therefore, the intermetallic phases Mg_51_Zn_20_ and MgZn_2_ appeared in the alloy at 200 °C and 405 °C, respectively. (Figure 3b,c) [33]. In addition, Ag can form the intermetallic phase Mg_3_Ag. It is interesting to note that after 24 h of milling, Ag, Zn and Mg, X-ray diffraction showed only the Mg pattern, and Ag and Zn disappeared in the spectra, which can be attributed to the elimination of elemental Ag and Zn. The Zn content in the alloy was 7 wt.%, and the Zn element mainly dissolves in primary Mg when the Zn content is less than 10 wt.%, which can improve the compressive strength of composites by increasing strength [34]. The presence of small amounts of elemental Ag can be helpful to maintain free ionic Ag in the physiological environment, which can improve bioactivity properties [35]. Peak intensity was comparable and the intensity of C1 was lower than C2 and C3 due to a smaller crystal size. In addition, increased imperfections, such as increasing dislocations due to the interaction between PCL particles and the alloy, can reduce the size of the crystals compared with C2 and C3 [36,37,38]. Moreover, the peaks of the composites were broader (due to the disordered chains) than those of the alloy, but the amorphous peaks were not observed due to the polymer content (5 wt.%). In C3, the difference between the XRD patterns demonstrates that the influence of Si occurs at 2ϴ = 53.66°, 2ϴ = 60.52° and 2ϴ = 82.31° [25]. According to the Mg–Zn binary alloy phase diagram [39], it is evident that metastable and intermetallic phases, such as Mg_7_Zn_3_ and Mg_2_Zn_3,_ certainly occurred at T > ~500 °C. In this study, an attempt was made to avoid these Mg_2_Zn_3_ and Mg_7_Zn_3_ residual phases and to secure a single stable MgZn phase in composites, taking into account the melting point of the polymers; one step was to use low temperatures for sintering. When taking into account the melting point of PVTMS and avoiding the formation of silicon carbide [40], the sintering temperature was set to 215 °C.

### 3.3. Study of Crystallite Size and TEM

**Figure 5:**
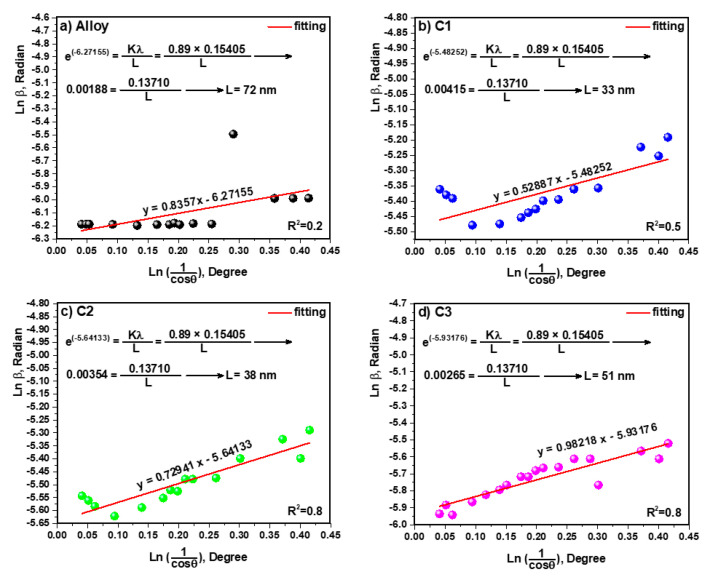
Linear plots of Monshi–Scherrer equation and calculated crystallite size of (**a**) Alloy, (**b**) C1, (**c**) C2 and (**d**) C3.

According to X-ray diffraction, the crystallite size values were calculated using the Monshi–Scherrer method and Equation (1) [41]. The linear plot of Ln β (radian) versus Ln $\left(\frac{1}{\cos\theta }\right)$ (degree) is established for all peaks of the alloy and composites (Figure 5). In Equation (1), β is the full width at half maximum (FWHM) of the peak in radians, K is the shape factor, usually estimated to be 0.89 for materials; λ is the wavelength of the radiation in nanometers $\lambda_{{\mathrm{Cu}}_{K\alpha }}$ = 0.15405 nm, ϴ is the diffraction angle of the peak, and L is the nanocrystal size [41,42].

(1) $$\mathrm{Ln}\;\beta =\mathrm{Ln}\;\left(\frac{K\lambda }{L}\right)+\mathrm{Ln}\;\left(\frac{1}{\cos\theta }\right)$$

The crystallite size values of 72, 33, 38 and 51 nm were gained for the alloy, C1, C2 and C3, respectively.

**Figure 6:**
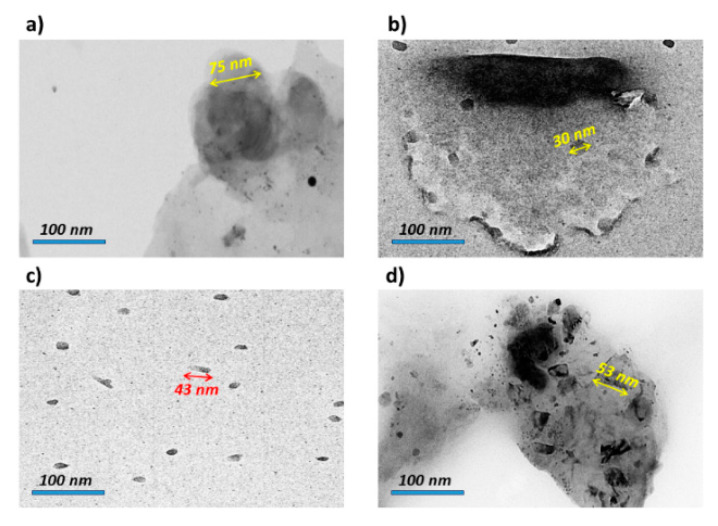
TEM images of (**a**) Alloy, (**b**) C1, (**c**) C2 and (**d**) C3.

**Figure 7:**
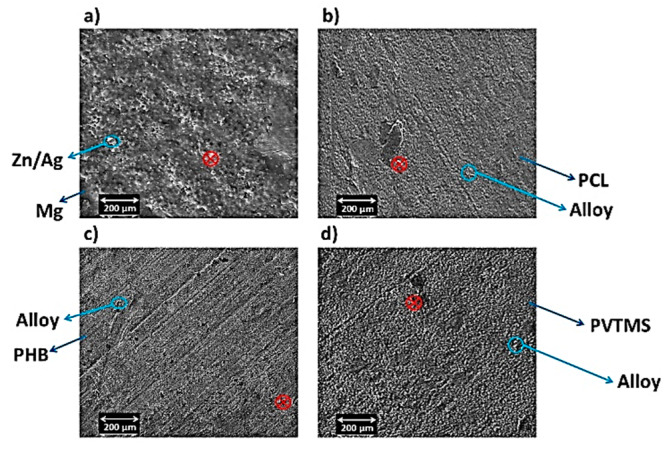
SEM images of (**a**) alloy, (**b**) C1, (**c**) C2 and (**d**) C3.

The TEM image of the alloy is shown in Figure 6a. In addition, the TEM images of the composites C1, C2 and C3 (Figure 7b–d) show the integration between the matrix and the reinforcement as an alloy. In this case, the reason for the formation of the nanocrystal structure is the fact that, during the mechanical alloy processing, the collision between balls and powders leads to a strong plastic deformation, and during the repetitive cycles of the powder, the particles break and combine with each other [43]. The crystal size of alloys and composites corresponded with the results extracted from the Monshi–Scherrer equation (X-ray diffraction). Additionally, the uniformity dispersion of polymers was observed, especially in Figure 6c. In Figure 6d, the crystal shapes in the dispersion of PVTMS were related to the effect of Si, so the carbide phase, such as SiC, can cause brittleness [44].

### 3.4. Study of SEM

**Figure 8:**
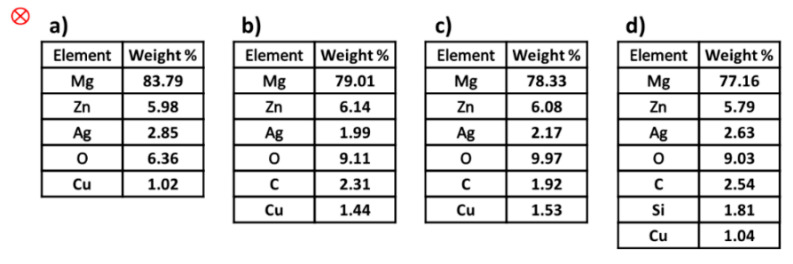
The extracted values of EDAX analysis: (**a**) alloy, (**b**) C1, (**c**) C2 and (**d**) C3.

Figure 7a shows the particles of the alloy and the non-uniformity occurring, which can be attributed to Mg_54_Ag_17_ and Mg_2_Zn_3_ [45], while in Figure 7b–d, the uniformity of the distribution of the particles increases by using polymers, causing a decrease in the light phase (white color). Moreover, the dispersion effect of the polymers is clearly observable when uniformity of distribution is performed. In addition, the homogeneous distribution of particles is increased when PHB is used as a reinforcement (Figure 7c). The images of SEM do not show any particular morphology and show two types of Mg-Zn-Ag particles: larger, elongated particles (white), and smaller, globular particles (black). Moreover, agglomeration and impressive porosity cannot be seen in the images. Even with the addition of silicon dioxide (derived from PVTMS), no fracture was observed, and the C3 composite requires more energy for fracture. Additionally, the intermetallic phases in the images of SEM were not observed in the composite samples, while the existence of intermetallic phases in the alloy samples is clear. An energy dispersive analysis X-ray (EDAX) was performed in a determined zone, and the extracted values from EDAX are listed in Figure 8. In this study, certain quantities of elements, it was found that amounts of elements such as magnesium (Mg), zinc (Zn), silver (Ag), oxygen (O), and copper (Cu as a source), were found in the area. It was observed that the Mg content in the composites decreased due to the use of polymers. It was also found that the weight percentages of O in the composites increased due to the high absorption of O. It is known that the weight percentage of silver in the composite samples decreased compared to the alloy, which can prevent the expansion of grain boundaries, and thus reduce the grain size.

### 3.5. Mechanical Properties

**Figure 9:**
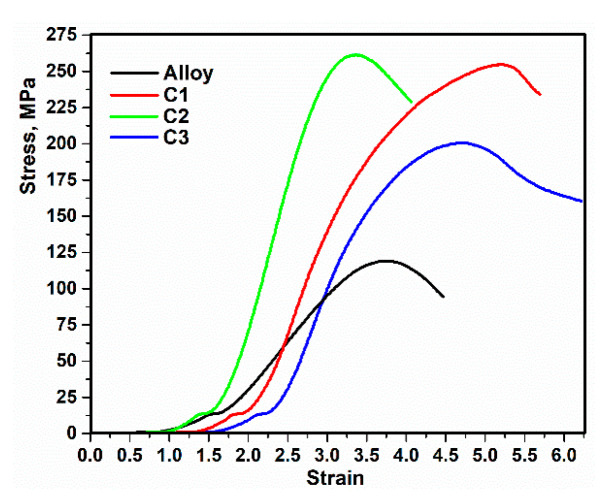
Strain–stress curves of alloy and composites.

Compressive strength test was carried out according to ASTM-E9 [46]. The stress–strain curves of alloy and composites are presented in Figure 9. The deformation at the beginning of the stress–strain curve (σ = ~14 MPa) is due to an increase in dislocation density, work hardening and the appearance of stiffness. Furthermore, the decrease in stress after loading is attributed to the work softening [47]. As can be seen, after the maximum tension ($\sigma_{\max}$), due to the increased activity of the slip–twinning systems and critical resolved shear stress, the strain increases and the stress decreases [48]. The mechanical properties of C1, C2, and C3 were improved, because the very low dissolution of PCL, PHB, and PVTMS in the alloy resulted in precipitation and dispersion around the grain boundaries. Therefore, the movement of the dislocations had become difficult and the onset of dynamic recrystallization was delayed [49]. Taking into account Equation (2), the slope of the stress–strain curve in the elastic region was used to calculate the elasticity coefficient [16]. Furthermore, in Equation (2), the σ and ε are the difference between two points of the stress and strain values, respectively.

(2) $$E=\frac{\sigma }{\varepsilon }$$

Taking into account ASTM-E384 [50], hardness values were calculated. The values extracted from the mechanical tests, consisting of maximum compressive strength, elasticity coefficient and hardness, are listed in Table 2. According to the values in Table 2, the results of the compressive strength test show that adding 5 wt.% PHB to the alloy can improve the mechanical compressive properties. Further, the presence of the polymers was effective, the size of the crystals was reduced, and the strength was increased. Moreover, these polymers prevent the creation of the brittle intermetallic phase. As can be seen from Table 2, the hardness of C2 is higher than that of the other composites and alloy, which is related to the decrease in the intermetallic phase. The charge transfers and dispersion of PHB in the alloy are more uniformly distributed than in C1 and C3, as well as showing the high proportion of locked dislocations and the formation of dislocation loops around the PHB particles, which led to an improvement in the mechanical properties of C2.

### 3.6. Hybrid Bonding between Alloy and Polymers

**Figure 10:**
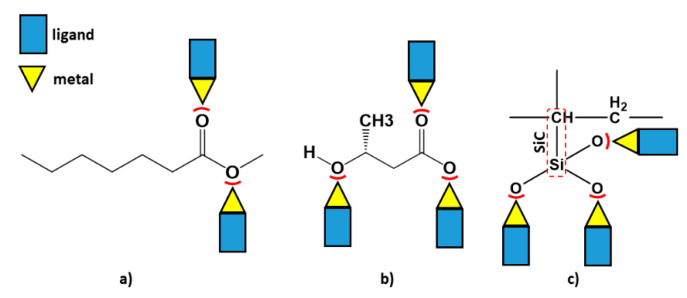
Coordination model of (**a**) C1, (**b**) C2 and (**c**) C3.

As shown in Figure 7b–d and the presence of oxygen and carbon (Figure 8), the aggregation of the polymers at the sintering temperatures resulted in hybrid bonding between organic and inorganic components. In these crystalline structures, non-covalent C-H⋯O type interactions provided the durability of structures through the bonding of alloy and polymers and formed a three-dimensional supramolecular network. Overall, intramolecular interactions increased aggregation. On the other hand, the mechanical properties of C2 were improved because the alloy Ag/Zn/Mg, based on PHB, exhibits self-aggregation reactions due to the large amount of oxygen atoms on its surface and its negative charge. These properties enable a variety of covalent, coordination and non-covalent interactions, such as electrostatic, van der Waals, hydrogen bonding and anion–π interactions. These interactions are the basis for the formation of an organic–inorganic hybrid structure based on PHB [51,52]. According to Figure 10b, the anions of PHB, which have many oxygen ends and bridges, can combine due to their coordination behavior. Furthermore, PVTMS in C3 created many bonds between oxygen and metal, but the presence of Si dominated the formation of silicon carbide, resulting in fragility and brittleness. According to Lindqvist law [53] a comparison of the power base of composites showed that the bridge oxygen of C2 had a greater power base than C1 and C2.

### 3.7. Investigation of Bioactivity

**Figure 11:**
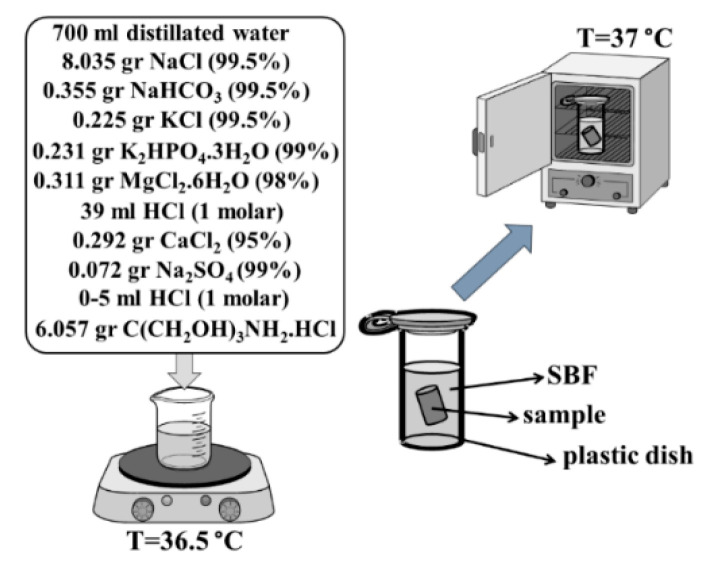
Synthesis route of SBF [54], immersed alloy and composites in the SBF at 37 °C.

According to the study by Kokubo et al. [54], solution body fluid (SBF) was synthesized. The alloy and composites were immersed in the SBF (Figure 11) and placed in an oven at 37 °C (similar to body temperature). After 5, 10, 20 and 30 days, the samples were removed and washed with distilled water. Analyses, such as XRD, FTIR, SEM and EDAX were thenconducted.

**Figure 12:**
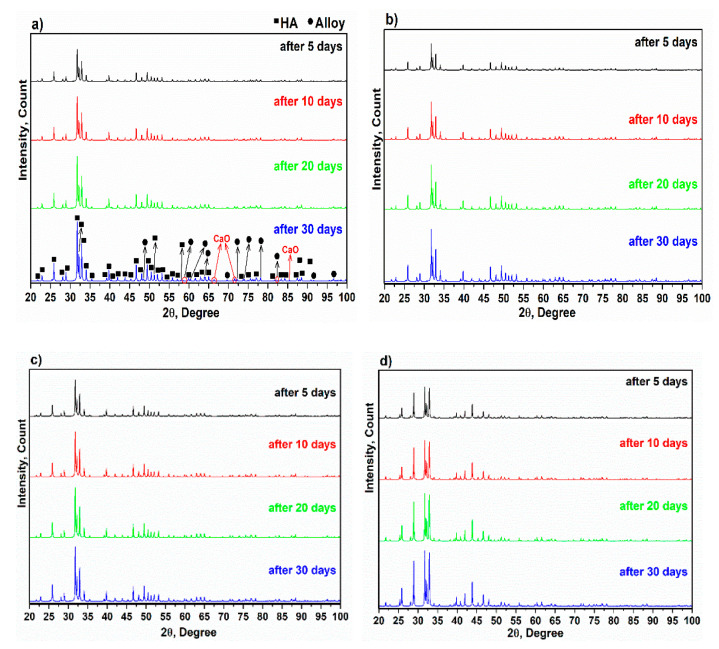
X-ray diffraction of immersed specimens in SBF after 5, 10, 20 and 30 days. (**a**) Alloy, (**b**) C1, (**c**) C2 and (**d**) C3.

One of the most important and essential features of nanocomposites for use as biomaterials in the body is the composition of hydroxyapatite on the surface [55]. The bioactivity of nanocomposites can be evaluated by their ability to form hydroxyapatite in SBF. Accordingly, the X-ray diffractions of alloy and composites immersed in SBF are shown in Figure 12. It is clear that the intensity of the XRD pattern increases after 5, 10, 20 and 30 days. Moreover, hydroxyapatite was formed, and the X-ray diffraction corresponds to those of the cited hydroxyapatite in Ref [9]. The X-ray diffraction of alloy and composites corresponded to standard JCPDS 9–432 [56]. The characteristic main peaks at ~2θ = 25.93°, 31.85° and 32.21° are related to planes (002), (211) and (300), respectively, showing that these angles and planes are directly attributed to hydroxyapatite [41,57]. Depending on the content of the alloy, the effect of the alloy, as well as the influence of CaO, is observed at ~2θ = 66.39° and 71.59° in tandem.

**Figure 13:**
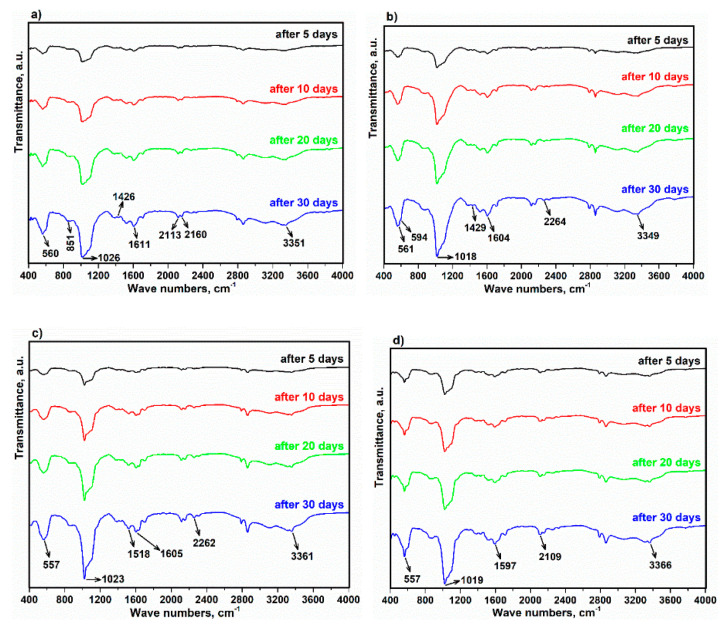
FTIR curves of immersed specimens in SBF after 5, 10, 20 and 30 days. (**a**) Alloy, (**b**) C1, (**c**) C2 and (**d**) C3.

Fourier transform infrared (FTIR) spectra of the alloy and composites immersed in SBF are shown in Figure 13. According to the FTIR spectrum, the group of ${\mathrm{PO}}_{4}^{3-}$ appeared at 560 and 851, 561 and 594, 557 cm^−1^ for the alloy and composites C1, C2 and C3, respectively. In addition, the strong stretching modes at 1026, 1018, 1023 and 1019 cm^−1^ are attributed to the ${\mathrm{PO}}_{4}^{3-}$ group. Evidence for the existence of carbonate groups in hydroxyapatite corresponded to wavenumbers at 1426 and 1611, 1429 and 1604, 1518 and 1605, and 1597 cm^−1^ for the alloy, and C1, C2, and C3 for composites in tandem. The stretching peaks in the range from 2109 to 2264 cm^−1^ are assigned to C–H [58]. Further, binding was found between O and H in the range from 3349 to 3361 cm^−1^ [59].

**Figure 14:**
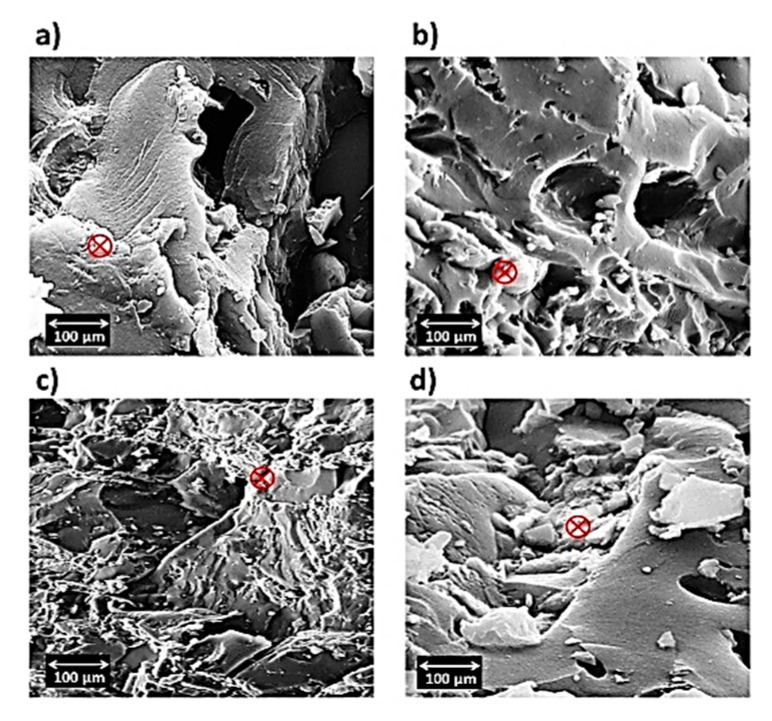
SEM images of immersed (**a**) alloy and composites (**b**) C1, (**c**) C2 and (**d**) C3 after 30 days.

**Figure 15:**
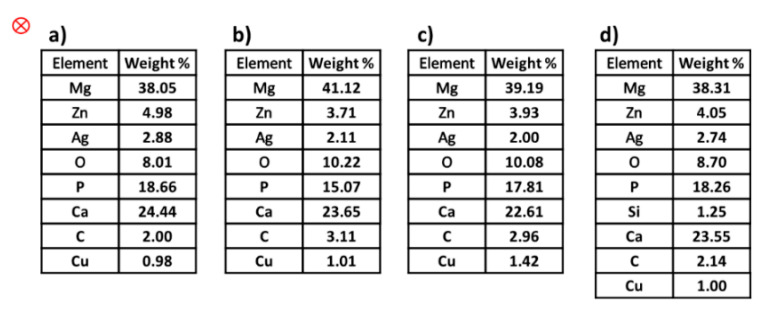
Extracted values from specific area of EDAX analysis of immersed (**a**) alloy and composites (**b**) C1, (**c**) C2 and (**d**) C3 after 30 days.

Following the investigation of the precipitation of hydroxyapatite on the alloy and composites after 5, 10, 20 and 30 days of immersion in SBF, SEM images were obtained. The images of the samples after 30 days’ immersion in SBF are shown in Figure 14. Hydroxyapatite formed and, after a few days, hydroxyapatite crystals grew. The images show that the light color of the pile figures indicates growing hydroxyapatite crystals, and the precipitation of hydroxyapatite continues. The alternative ratio of calcium to phosphate plays a very important role in the bioactivity properties. Furthermore, the EDAX analysis of these samples (after immersion in SBF) was studied, and the extracted values are tabulated in Figure 15. Taking into account the ratio of the weight percentage of calcium (Ca) to phosphorus (P), these values were calculated as 1.30, 1.56, 1.26 and 1.28 for alloy, and composites C1, C2 and C3, respectively. The base ratio of Ca/P for the presence of hydroxyapatite is 1.67 [60], and the value of C2 is closer to this ratio than the other samples.

## 4. Conclusions

The alloy, consisting of 3 wt.% Ag/7 wt.% Zn/90 wt.% Mg, was well-prepared by a mechanical alloying process. Furthermore, the effect of 5 wt.% polymers, consisting of PCL, PHB and PVTMS, on the alloy was investigated. The thermal properties of composites were evaluated by DSC studies, and the melting points of the polymers, PCL, PHB and PVTMS were estimated to be 57 °C, 149 °C and 223 °C, respectively, which resulted in the sintering temperature of the composites being considered lower than the melting point of the polymers to prevent collapse. The X-ray diffraction of alloy and composites was studied, and the intermetallic phases decreased when the polymers were added to the alloy, which was attributed to the dispersion of polymers. In addition, the crystallite size values were calculated using the Monshi–Scherrer method, and values of 72, 33, 38 and 51 nm were obtained for the alloy, C1, C2 and C3, respectively. The extracted values from the Monshi–Scherrer method were confirmed by the TEM analysis. Moreover, the SEM images showed the intermetallic phase for the alloy and the uniformity distributions for the composites. The mechanical properties were discussed in detail. The presence of PHB in the alloy led to an improvement in mechanical strength because the hybrid strength of C2 was stronger than that of C1 and C3 due to the connection of oxygen with the metal. The alloy and composites were bioactive when the hydroxyapatite crystals were precipitated on the surface of samples after immersion in SBF. In addition, the Ca/P ratio of the samples was in the range from 1.26 to 1.56 and the ratio of composites containing PHB was closer to that of natural hydroxyapatite (1.67).

## Figures and Tables

**Table 1:** The theoretical and experimental values of density and porosity.

Sample	$\rho_{\mathrm{theoretical}},$ (gr/cm^3^)	$\rho_{\mathrm{experimental}},$ (gr/cm^3^)	$\frac{\rho_{\mathrm{experimental}}}{\rho_{\mathrm{theoretical}}}$	Porosity, (%)
**Alloy**	1.792 ± 0.005	1.721 ± 0.003	0.96038	4.610 ± 0.008
**C1**	1.613 ± 0.007	1.608 ± 0.008	0.9969	5.011 ± 0.006
**C2**	1.779 ± 0.001	1.711 ± 0.003	0.96178	5.063 ± 0.009
**C3**	1.783 ± 0.003	1.701 ± 0.004	0.95401	4.917 ± 0.005

**Table 2:** The values of compressive strength, elasticity coefficient and hardness of alloy and composites.

Sample	Maximum Compressive Strength, (MPa)	Elasticity Coefficient, (MPa)	Hardness, (HV)
**Alloy**	119.05 ± 1.03	65.45 ± 1.01	57.39 ± 1.00
**C1**	255.39 ± 1.11	134.25 ± 1.00	68.71 ± 1.01
**C2**	261.84 ± 1.08	228.88 ± 1.03	71.05 ± 1.01
**C3**	201.61 ± 1.14	134.84 ± 1.00	60.26 ± 1.00

## Data Availability

Data sharing is not applicable.

## Supplementary Materials

The following supporting information can be downloaded at: https://www.mdpi.com/article/10.3390/ma15155421/s1. Figure S1. The structure of PCL; Figure S2. The structure of PHB; Figure S3. The structure of PVTMS; Figure S4. The schematic route for preparing an Ag/Zn/Mg alloy.
