# Supplementary material for: Effects of 5 wt.% Polycaprolactone, Polyhydroxybutyrate and Polyvinyltrimethoxysilane on the Properties of Ag/Zn/Mg Alloy
Source: Materials (Basel). 2022 Aug 5;15(15):5421. doi: 10.3390/ma15155421 (PMC9369843; doi:10.3390/ma15155421)
Supplement: Supplementary file 1 [file materials-15-05421-s001.zip › materials-1808440-supplementary.pdf]

materials

MDPI

Supplementary Materials

Effects of 5 wt.% Polycaprolactone, Polyhydroxybutyrate and Polyvinyltrimethoxysilane on the Properties of Ag/Zn/Mg Alloy

Marzieh Rabiei [1], Motahareh Sadat Raziyan [1], Reza Ebrahimi-Kahrizsangi [2], Sohrab Nasiri [1,*], Arvydas Palevicius [1], Giedrius Janusas [1], and Andrius Vilkauskas [1]

Materials and Instruments

Silver powder (99.9%), zinc powder (98.8%) and magnesium powder (98.8%) Sigma Aldrich were prepared. In addition, Polycaprolactone (PCL) and PolyHydroxyButyrate (PHB) Sigma Aldrich; Mw = 80,000 g/mol were prepared. In addition, vinyltrimethoxysilane (VTMS) (Merck) was chosen in this study. In addition, differential scanning calorimetry (DSC) were carried out by STA-BAHR and the rate of heating was set at of 5 °C/min. Powders X-Ray, phase series were confirmed by X-ray diffraction (XRD) and performed on a Philips XRD diffractometer, Cuk_{α} radiation was used at 40 KV, 30 mA, step size of 0.05° (2Ɵ) nd scan rate of 1°/min (AXS GmbH, Kaunas, Lithuania). Furthermore, X'Pert software was used for qualitative analysis and report of width diffraction peaks (rad, β) at full width half maximum (FWHM) in different 2θ values according to the situation of peaks (Version 4.9.0). Moreover, transmission electron microscopy (TEM) Tecnai G2 F20 X–TWIN with acceleration voltage from 50 to 80 KV was utilized. For chemical elements of components, an energy dispersive X-Ray (EDX) spectrometer Phillips/FEI 149 Quanta 200 was utilized (Iran, Tehran). In addition, scanning electron microscope analysis (SEM) Phillips/ FEI Quanta 200 was used to study. Fourier-transform infrared spectroscopy (FTIR) spectra of the compounds was attached in the potassium bromide (KBr) powders and the instrument that was used was a Perkin-Elmer Spectrum BX FT-IR spectrometer (Iran, Isfahan). the morphology of the compounds and scaffold. In addition, for investigating the mechanical properties, compressive strength test was carried out according to ASTM-E9 on the specimens at room temperature. Cylindrical specimens with a diameter of 5 mm and a height of 10 mm were prepared. The relative velocity of the movement of the jaws was assumed to be 0.1 mm per minute. In addition, to calculate the hardness values of each samples, five points of the specimens were examined and their average was considered as the hardness value. In addition, simulated body fluid (SBF) was prepared according to the method of Kokubo et al. [1]. Considering the temperature at 37 °C, materials were added and dissolved according to the Kokubo method. Afterward, $(CH_2OH)_3CNH_2$ and HCl (merck) were added (dropwise) to achieve a pH of 7.40 (final pH), then the temperature was decreased (20 °C) and distilled water was added [1].

PCL

PCL is a polymer composed of hexanoate repeating units and belongs to the class of aliphatic polyesters. PCL is a synthetic, biodegradable polymer approved for using as a bone graft substitute [2]. The chemical formula of PCL is $(C_6H_{10}O_2)_n$. PCL is highly hydrophobic, semi-crystalline, highly soluble at room temperature, and easy to process due to has low melting temperature and good biocompatibility thus researchers investigated potential applications, especially in the biomedical fields [3]. The physical, thermal

and mechanical properties of PCL depend mainly on molecular weight and degree of crystallinity, which also contribute to degrade under physiological conditions by hydrolysis of its ester bonds [4]. Furthermore, the mechanical and bioactive properties of a PCL scaffold can be improved by adding bioactive materials such as bio-metals [2]. Previous studies on PCL have shown that the destruction rate is very slow and PCL can maintain mechanical properties longer than other biopolymers [5],[6]. In 2017, Jing et al. prepared composite scaffolds of PCL/hydroxyapatite [7]. Moreover, Goh et al. used PCL as an ingredient for the fabrication of scaffolds for bone tissue engineering [8]. PCL is an aliphatic polyester and a semi-crystalline polymer with a degree of crystallinity up to 69%. The unit cell is orthorhombic and the lattice constants are a = 7.49 A˚, b = 4.97 A˚ and c = 17.29 A˚ [9]. Most of the applications of PCL are related to tissue engineering due to suitable mechanical properties and creation of porosity with suitable area [5]. The chemical structure of PCL is shown in **Figure S1**.

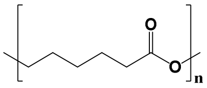

Figure S1. The structure of PCL.

PHB

PHB is a linear polyester of D(-)-3-hydroxybutyric acid whose structure was first discovered in 1925 by Anderson et al. [10]. PHB is a polymer belonging to the class of polyesters, which are interest as biologically produced and biodegradable plastics. PHB is a polymer that is insoluble in water, resistant to ultraviolet radiation and impermeable to oxygen. PHB has shown a good degree of biocompatibility with various cells and can be produced by many species of microorganisms [11]. PHB has exceptional stereo chemical purity and is fully isotactic and crystallizable, but its brittleness and rigidity limit its applications [12]. PHB has low toxicity due in part to its in vivo fragility or transformability. Senatov et al. used PHB in a hydroxyapatite scaffold and demonstrated that PHB is bioactive and suitable for increasing the ratio of bioactivity [13]. Costad et al. investigated the bioactivity of glass/PHB composites and the biocompatibility of PHB was proven [14]. The chemical structure of PHB is shown in **Figure S2**. PHB is derived from polyhydroxyalkanoate, which is a linear polymer of 3-hydroxybutyrate. PHB is the most abundant type of monomer prepared from a short chain [15]. The physical and chemical properties of PHB are similar to those of polyethylene and polypropylene. The chemical formula is $(C_4H_6O_2)_n$ [16].

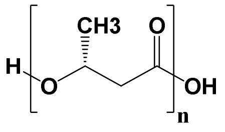

Figure S2. The structure of PHB.

PVTMS

PVTMS is synthesized by polymerization of VTMS [17]. The features of vinyl group (–CH–CH_2) in the structure of PVTMS can be used to perform free radical polymerization. Moreover, PVTMS can improve the thermal stability and physical properties of the scaffold [18]. Moreover, Rabiei et al, has proven that PVTMS is bioactive [17],[19]. It has a functional group like silanol (Si-O-H) that can help with bonding; therefore, it is useful for preventing the decomposition of composites [18]. PVTMS is a polysiloxane-based component that is particular interest due to its dense structure of siloxane cross-linked with polymeric groups. In addition, PVTMS improves mechanical properties by forming a stable Si-O-Si framework [20]. PVTMS is an organo-silane molecule and provides a hydrophobic environment in the composite. Since water can easily diffuse into the

polymer structure, it can lead to the breakdown of intermolecular forces and create voids in the polymer [21]. The chemical structure of PVTMS is presented in **Figure S3**.

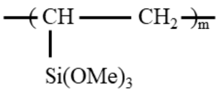

Figure S3. The structure of PVTMS.

Preparation of Ag/Zn/Mg alloy

The schematic way to prepare the new Ag/Zn/Mg alloy is shown in **Figure S4**. 1) To prepare the alloy, powders consisting of 3 wt.% Ag/7 wt.% Zn/90 wt.% Mg with a certain weight percentage, were mixed in a high-energy planetary ball mill. Taking into account amount of powder and sufficient space for transferring the energy from the balls to the powder, the beaker was filled to 30%. In this case, hard steel enclosures (V=250 ml) and steel balls (D=20 mm) were used. The rate of enclosures was adjusted 250 rpm and to prevent temperature rise, 10 minutes was considered as rest time after 30 minutes of system operation. Moreover, Mg is very active in an oxygenated environment; therefore, argon (Ar) was chosen as the gas to cover the enclosures, reducing the oxygen content to ≤ 3 ppm. 2) Taking into account the height to diameter ratio (1.7), the force of the jaws was set to 88×10^7 pa and a cylindrical specimen was fabricated. 3) For the sintering process, the sample was placed in the furnace and according to the sensitivity of metals, especially Mg, to oxygen; Ar gas was blowed into the environment of the furnace. The rate of the temperature was slow (5 °C/min) and the temperature of the furnace was constant at 199 °C for 2 hours and 405 °C for 2 hours tandemly.

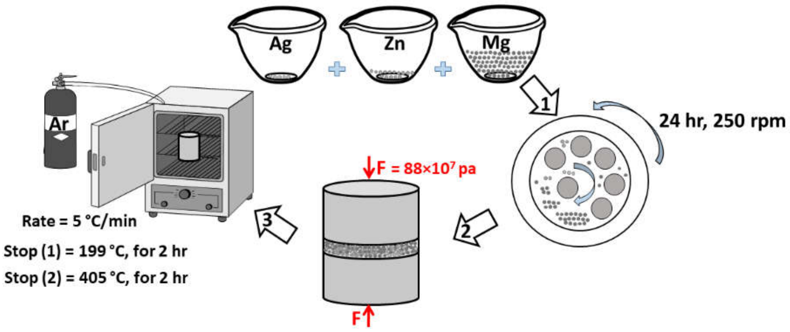

Figure S4. The schematic route for preparing an Ag/Zn/Mg alloy.

References

1. Kokubo, T., and Takadama, H. (2006) How useful is SBF in predicting in vivo bone bioactivity? *Biomaterials*, **27** (15), 2907–2915.
2. Chuenjitkuntaworn, B., Inrung, W., Damrongsri, D., Mekaapiruk, K., Supaphol, P., and Pavasant, P. (2010) Polycaprolactone/hydroxyapatite composite scaffolds: Preparation, characterization, and in vitro and in vivo biological responses of human primary bone cells. *J. Biomed. Mater. Res. - Part A*, **94** (1), 241–251.
3. Nair, L.S., and Laurencin, C.T. (2007) Biodegradable polymers as biomaterials. *Prog. Polym. Sci.*, **32** (8–9), 762–798.
4. Lowery, J.L., Datta, N., and Rutledge, G.C. (2010) Effect of fiber diameter, pore size and seeding method on growth of human dermal fibroblasts in electrospun poly(ε-caprolactone) fibrous mats. *Biomaterials*, **31** (3), 491–504.
5. Rezwan, K., Chen, Q.Z., Blaker, J.J., and Boccaccini, A.R. (2006) Biodegradable and bioactive porous polymer/inorganic composite scaffolds for bone tissue engineering. *Biomaterials*, **27** (18), 3413–3431.
6. Gunatillake, P.A., Adhikari, R., and Gadegaard, N. (2003) Biodegradable synthetic polymers for tissue engineering. *Eur. Cell. Mater.*, **5**, 1–16.

7. Jing, X., Mi, H.Y., and Turng, L.S. (2017) Comparison between PCL/hydroxyapatite (HA) and PCL/halloysite nanotube (HNT) composite scaffolds prepared by co-extrusion and gas foaming. *undefined*, **72**, 53–61.
8. Goh, C.S., Gupta, M., Jarfors, A.E.W., Tan, M.J., and Wei, J. (2007) A Novel Polycaprolactone/Hydroxyapatite Scaffold for Bone Tissue Engineering. *Key Eng. Mater.*, **342–343**, 265–268.
9. Hu, H., and Dorset, D.L. (1990) Crystal Structure of Poly(ϵ-caprolactone). *Macromolecules*, **23** (21), 4604–4607.
10. Anderson, A.J., and Dawes, E.A. (1990) Occurrence, metabolism, metabolic role, and industrial uses of bacterial polyhydroxyalkanoates. *Microbiol. Rev.*, **54** (4), 450–472.
11. Jacquel, N., Lo, C.W., Wei, Y.H., Wu, H.S., and Wang, S.S. (2008) Isolation and purification of bacterial poly(3-hydroxyalkanoates). *Biochem. Eng. J.*, **39** (1), 15–27.
12. Vergara-Porras, B., Pérez-Guevara, F., and Gracida-Rodríguez, J.N. (2016) Study of thermal, crystalline, tensile and biodegradation properties of 70/30 poly(β-hydroxybutyrate/poly(ε-caprolactone) melt-mixed blends: the influence of compression molding conditions. *N. Biotechnol.*, **33** (3), 428.
13. Senatov, F., Anisimova, N., Kiselevskiy, M., Kopylov, A., Tcherdyntsev, V., and Maksimkin, A. (2017) Polyhydroxybutyrate/Hydroxyapatite Highly Porous Scaffold for Small Bone Defects Replacement in the Nonload-bearing Parts. *J. Bionic Eng.*, **14** (4), 648–658.
14. Oliveira Paiva, A., Duarte, M.G., Helena, M., Fernandes, V., Gil, M.H., and Gomes Costa, N. (2006) In vitro studies of bioactive glass/polyhydroxybutyrate composites. *SciELO Bras.*, **9** (4), 417–423.
15. Sharma, L., Kumar Singh, A., Panda, B., and Mallick, N. (2007) Process optimization for poly-β-hydroxybutyrate production in a nitrogen fixing cyanobacterium, Nostoc muscorum using response surface methodology. *Bioresour. Technol.*, **98** (5), 987–993.
16. Panda, B., Jain, P., Sharma, L., and Mallick, N. (2006) Optimization of cultural and nutritional conditions for accumulation of poly-beta-hydroxybutyrate in Synechocystis sp. PCC 6803. *Bioresour. Technol.*, **97** (11), 1296–1301.
17. Rabiei, M., Palevicius, A., Ebrahimi-Kahrizsangi, R., Nasiri, S., Vilkauskas, A., and Janusas, G. (2021) New Approach for Preparing In Vitro Bioactive Scaffold Consisted of Ag-Doped Hydroxyapatite + Polyvinyltrimethoxysilane. *Polymers (Basel).*, **13** (11), 1695.
18. Lim, M., Kim, D., Seo, J., and Han, H. (2014) Preparation and properties of poly(vinyl alcohol)/vinyltrimethoxysilane (PVA/VTMS) hybrid films with enhanced thermal stability and oxygen barrier properties. *Macromol. Res.*, **22** (10), 1096–1103.
19. Nasiri, S., and Nasr-Esfahani, M. (2013) Bioactive Organic-Inorganic Composite Monolith Derived from Poly Vinyl Trimethoxy Silane Using Sol- Gel Process. *undefined*.
20. Motalebi, A., Nasr-Esfahani, M., Ali, R., and Pourriahi, M. (2012) Improvement of corrosion performance of 316L stainless steel via PVTMS/henna thin film. *Prog. Nat. Sci. Mater. Int.*, **22** (5), 392–400.
21. Lewis, H.G.P., Casserly, T.B., and Gleason, K.K. (2001) Hot-Filament Chemical Vapor Deposition of Organosilicon Thin Films from Hexamethylcyclotrisiloxane and Octamethylcyclotetrasiloxane. *J. Electrochem. Soc.*, **148** (12), F212.
